# Buckwheat Cakes

**Published:** 1856-12

**Authors:** 


BUCKWHEAT CAKES.—To every three bushels of buckwheat, add one of good heavy oats; grind them together as if there was only buckwheat; thus will you have cakes always light and always brown, to say nothing of the greater digestibility, and the lightening of spirits, which are equally certain.

